# Low-dose Paclitaxel with Pembrolizumab Enhances Clinical and Immunologic Responses in Platinum-refractory Urothelial Carcinoma

**DOI:** 10.1158/2767-9764.CRC-23-0436

**Published:** 2024-02-26

**Authors:** Rhonda L. Bitting, Janet A. Tooze, Michael Goodman, Donald C. Vile, Jessica M. Brown, Christopher Y. Thomas, Morgan Neve, Mitra Kooshki, Safoa Addo, Pierre L. Triozzi, Purnima Dubey

**Affiliations:** 1 Department of Internal Medicine, Section on Hematology and Oncology, Wake Forest School of Medicine, Winston-Salem, North Carolina.; 2 Wake Forest Baptist Comprehensive Cancer Center, Winston-Salem, North Carolina.; 3 Department of Biostatistics and Data Science, Wake Forest School of Medicine, Winston-Salem, North Carolina.; 4 Department of Microbial Infection and Immunity, The Ohio State University, Columbus, Ohio.; 5 Pelotonia Institute of Immunooncology, James Comprehensive Cancer Center, The Ohio State University, Columbus, Ohio.

## Abstract

**Purpose:**

Single-agent checkpoint inhibition is effective in a minority of patients with platinum-refractory urothelial carcinoma; therefore, the efficacy of combining low-dose paclitaxel with pembrolizumab was tested.

**Materials and Methods:**

This was a prospective, single-arm phase II trial with key inclusion criteria of imaging progression within 12 months of platinum therapy and Eastern Cooperative Oncology Group ≤1. Treatment was pembrolizumab 200 mg day 1 and paclitaxel 80 mg/m^2^ days 1 and 8 of a 21-day cycle for up to eight cycles unless progression or unacceptable adverse events (AE). The primary endpoint was overall response rate (ORR) with overall survival (OS), 6-month progression-free survival (PFS), and safety as key secondary endpoints. Change in circulating immune cell populations, plasma, and urinary miRs were evaluated.

**Results:**

Twenty-seven patients were treated between April 2016 and June 2020, with median follow-up of 12.4 months. Baseline median age was 68 years, with 81% men and 78% non-Hispanic White. ORR was 33% by intention to treat and 36% in imaging-evaluable patients with three complete responses. Six-month PFS rate was 48.1% [95% confidence interval (CI): 28.7–65.2] and median OS 12.4 months (95% CI: 8.7 months to not reached). Common ≥ grade 2 possibly-related AEs were anemia, lymphopenia, hyperglycemia, and fatigue; grade 3/4 AEs occurred in 56%, including two immune-mediated AEs (pneumonitis and nephritis). Responding patients had a higher percentage of circulating CD4^+^IFNγ^+^ T cells. Levels of some miRs, including plasma miR 181 and miR 223, varied in responders compared with nonresponders.

**Conclusions:**

The addition of low-dose paclitaxel to pembrolizumab is active and safe in platinum-refractory urothelial carcinoma.

**Significance:**

We found that combining pembrolizumab with low-dose paclitaxel may be effective in patients with urothelial carcinoma progressing on platinum chemotherapy, with favorable safety profiles.

## Introduction

Multiple checkpoint inhibitors are approved for the treatment of metastatic urothelial cancer (1–3). In the post-platinum setting, pembrolizumab, nivolumab, and avelumab are all approved, with pembrolizumab listed as category 1 by national comprehensive cancer network (NCCN) given a phase III trial demonstrating a 3-month improvement in overall survival (OS) over chemotherapy (1). Overall response rates (ORR) are approximately 20%, yet responses can be durable, begging the question of how the benefits of immunotherapy can be extended to more people. The answer may lie in increasing the antitumor immune responses through manipulation of the tumor, its stroma, and the surrounding immune infiltrate.

The interaction between PD-1 and its ligand, PD-L1, leads to T-cell anergy, allowing tumor cells to evade detection and destruction (4). Disrupting the interaction between PD-1, expressed on activated cytotoxic CD8^+^ T cells, and PD-L1, expressed on tumor cells, T regulatory (T_REG_) cells, and tumor-associated macrophages, results in activation and proliferation of antigen-experienced cytotoxic T cells present in the tumor microenvironment (4, 5). Some studies report that CD4^+^IFNγ^+^ T cells also have cytolytic function and contribute to antitumor responses (6, 7). Resistance to PD-1 inhibition may be due to the lack of or impaired presentation of tumor antigens, lack of immune infiltration into the tumor, increase in immune suppressive cells such as myeloid-derived suppressor cells (MDSC), alternate immune checkpoints, or T-cell exhaustion or epigenetic changes (5). Overcoming these mechanisms of resistance is critical, and combining checkpoint inhibition with other agents is a next logical step and the topic of a large body of ongoing research. The excellent efficacy shown when pembrolizumab is combined with the antibody–drug conjugate enfortumab vedotin (EV), for example, illustrates the potential for improved outcomes over single-agent PD-1 inhibition (8).

Although chemotherapy was initially assumed to be an antagonistic combination with immunotherapy due to its lymphodepleting effects, it has proven to be a promising partner, particularly in non–small cell lung cancer (NSCLC), where pembrolizumab plus chemotherapy has demonstrated superiority to chemotherapy alone (9). The results of chemoimmunotherapy in bladder cancer, however, have been disappointing. In the IMvigor130 phase III trial, the addition of atezolizumab to first-line chemotherapy for metastatic urothelial cancer demonstrated an improvement in progression-free survival (PFS) compared with chemotherapy alone (10). However, the KEYNOTE-361 study which compared platinum-based chemotherapy alone versus pembrolizumab plus chemotherapy in the first-line metastatic setting did not meet its dual primary endpoint of improved PFS and OS, suggesting that work is needed to refine the patient population and/or combination therapy choices (11).

Chemotherapy may synergize with PD-1/PD-L1 inhibition via induction of tumor cell death, resulting in autovaccination through the release of tumor cell antigens, and through immunomodulation of the tumor microenvironment, thereby disrupting the strategies that tumors use to evade the immune response (12). The immunomodulatory effects of chemotherapy are dose and time dependent. In preclinical studies, low-dose paclitaxel upregulates dendritic cells to present antigens to antigen-specific T cells (13) and selectively impairs T_REG_ cells while sparing T effector cells (14, 15). Furthermore, paclitaxel reduces tumor-associated immunosuppression via a decrease in the quantity and function of MDSCs, and a decrease in immunosuppressive factors including TGFβ, CCL2, VEGF, CCL22, COX-2, and IL10 (15–17). Given its demonstrated immunomodulatory effects on several possible pathways of resistance to PD-1/PD-L1 inhibition, paclitaxel may be an ideal addition to anti-PD-1/PD-L1 therapy. Furthermore, urothelial cancer should be a prime candidate for combination chemoimmunotherapy, as the urothelial cancer tumor microenvironment is dominated by T_REG_ cells and immune inhibitory cytokines. (18). In addition, MDSCs with potent T-cell suppressive activities are increased in urothelial cancer (19, 20). Although PD-1 blockade partially overcomes MDSC and T_REG_ suppression in tumor models (21), the treatment effect of immune checkpoint inhibitors is maximized when MDSCs are fully eradicated (22). Urothelial cancer preclinical studies similarly show that abrogating immune suppression maximizes the antitumor efficacy of PD-1 blockade (23). Therefore, the efficacy of PD-1/PD-L1 inhibition may be enhanced in urothelial cancer when given in combination with agents that augment antitumor immune responses.

In this study, we evaluated the efficacy of low-dose paclitaxel and pembrolizumab in patients with platinum-refractory, metastatic urothelial cancer in a single-arm, phase II trial. We hypothesized that combination therapy would improve the ORR relative to single agent pembrolizumab alone. We also looked at potential biomarkers at baseline, during, and at completion of therapy, including the number and proportion of CD8^+^IFNγ^+^ and CD4^+^IFNγ^+^ T cells, MDSCs, and T_REG_s, and changes in plasma and urine immune-regulatory miRNAs, to identify patients who may benefit from combination chemoimmunotherapy in urothelial cancer.

## Materials and Methods

### Patients

This was a prospective, single-arm phase II trial (NCT02581982) conducted at Wake Forest University Comprehensive Cancer Center (Winston Salem, NC), with patients recruited between April 2016 and June 2020. The study was approved by the Wake Forest Health Sciences Institutional Review Board, and all patients provided written informed consent before undergoing any study-related procedures or testing. Eligible patients were ≥18 years old with an Eastern Cooperative Oncology Group (ECOG) performance status score of <2 and histologically- or cytologically-confirmed urothelial carcinoma of the renal pelvis, ureter, bladder, or urethra, of predominantly transitional cell type. Participants had metastatic platinum-refractory disease, defined as disease progression on or within 12 months of cisplatin or carboplatin therapy, with radiographic progression prior to study entry with at least one measurable lesion as per RECIST version 1.1. Key exclusion criteria included more than two previous lines of systemic treatment; active, untreated central nervous system metastases; previous exposure to PD-1/PD-L1 inhibitors or taxane chemotherapy; history of pneumonitis; infection with human immunodeficiency virus or hepatitis B/C; pregnancy; requirement for ongoing immunosuppression; and active uncontrolled infection. The study design is illustrated in Supplementary Fig. S1.

### Treatment

Patients were treated with pembrolizumab 200 mg on day 1 and paclitaxel 80 mg/m^2^ on days 1 and 8 of a 21-day cycle for up to eight cycles unless clinical or radiographic disease progression or unacceptable adverse events (AE) were observed. Patients with stable or improved disease at the end of combination therapy could remain on pembrolizumab maintenance for up to 2 years. There were no pembrolizumab dose adjustments, though treatment could be held for up to 12 weeks if necessary for management of immune-related AEs. Standard dose adjustments were used for paclitaxel.

### Study Assessments

Baseline assessments included a complete medical history, physical exam, complete blood count, comprehensive metabolic panel, lactate dehydrogenase, thyroid studies, and a CT scan of the chest, abdomen, and pelvis. Patients were clinically assessed on day 1 of each cycle and were evaluated for response by imaging every three cycles or sooner if clinical suspicion for disease progression. Patients who received at least one dose of pembrolizumab with paclitaxel and did not have subsequent restaging imaging were not evaluable for the primary study endpoint of ORR. Baseline characteristics and toxicity data were reported on all participants. Similarly, all participants were included in the intention-to-treat analyses including PFS and OS. Complete response (CR), partial response (PR), stable disease (SD), and progressive disease (PD) were evaluated and assigned according to RECIST version 1.1. Patients with CR, PR, or SD were considered to have achieved disease control. All imaging assessments were reviewed by at least one investigator and radiologist. AEs were characterized and graded using Common Terminology Criteria for Adverse Events version 4.0. The relatedness between treatment and AEs was determined by the treating physician and investigator.

### Correlative Assessments

#### Tumor Tissue for PD-L1 Testing

Tumor tissue was collected via metastatic biopsy or by accession of archival tissue obtained within 6 months of treatment on study. Five unstained ProbeOn Plus slides per patient, cut from a formalin-fixed, paraffin-embedded tissue block, were sent to Discovery Life Sciences (formerly QualTek Molecular Labs) for PD-L1 staining. As per the Merck Investigator Study Program, PD-L1 membrane expression was measured on tumor cells and mononuclear inflammatory cells (MIC) using the anti-PD-L1 antibody 22C3 as described previously (24). Results were reported as a modified proportion score (MPS), which is the overall % positive cells expressing PD-L1, inclusive of both tumor and tumor-infiltrating MICs (24).

#### Blood and Urine for Immune Correlates

Blood and urine were collected prior to treatment administration on day 1 of cycle 1, on day 1 of cycle 4, on day 1 of cycle 7, and at the end of combination treatment. Ten mL of blood were drawn into ethylenediamine tetraacetic acid (EDTA) tubes, and peripheral blood mononuclear cells (PBMC) and plasma were separated by gradient centrifugation. Urine was centrifuged at 1,000 × *g* for 10 minutes to pellet cellular debris. All samples were aliquoted and stored at −80°C until further use.

To evaluate changes in effector T-cell number and function, PBMCs were thawed, counted, and plated in T-cell media overnight. The next day, the cells were stimulated with phorbol 12-myristate 13-acetate/ionomycin for 5 hours, and then stained with antibodies specific for the cell surface markers CD8, CD4, CD25, CD14, HLA-DR. Cells were then fixed, permeabilized, and stained with anti-IFNγ antibody to ascertain function. A subset of cells was stained intracellularly with FoxP3. Cells were evaluated by multicolor flow cytometry using a FACSCalibur flow cytometer (BD Biosciences). Immune cell populations were identified using phycoerythrin-labeled FoxP3 and HLA-DR, FITC-labeled CD4 and CD14. All labeled antibodies were purchased from BD Biosciences except for FoxP3, which was from eBiosciences. The percentage of populations of interest was determined using gate statistics. The absolute number of MDSCs was calculated as follows: [total white blood cell count (cells/µL) × percent MDSCs]. T_REG_ cell frequencies were calculated by normalization to total CD4 T-cell numbers.

For analysis of immunomodulatory miRNA, total RNA was isolated from plasma and urine using the miRNeasy Mini kit (Qiagen Inc.) according to the manufacturer's instructions. Reverse transcription reactions were performed using a TaqMan MicroRNA Reverse Transcription kit (Applied Biosystems; Thermo Fisher Scientific, Inc.) per manufacturer's instructions. qRT-PCR was performed using the reverse transcription reaction product, TaqMan MicroRNA Assay kit, and TaqMan Universal PCR Master Mix (Applied Biosystems; Thermo Fisher Scientific, Inc.) according to the manufacturer's instructions. TaqMan MicroRNA Assay kits for human miRs were used, including miR 20a, miR 21, miR 125, miR 146a, miR 155, miR 181, and miR 223. Reactions were loaded onto a 96-well plate and run in duplicate on an ABI 7500 Fast Real-Time PCR System (Applied Biosystems; Thermo Fisher Scientific, Inc.). The reactions were incubated at 50°C for 20 seconds and 95°C for 10 minutes, followed by 40 cycles of denaturation at 95°C for 15 seconds, then 1 minute of annealing/extension at 60°C. The ΔΔCt method was used to determine relative number of copies [relative quantification (RQ)] of the miRNA (miR). Data were normalized to a C. elegans synthetic miR sequence, cel-miR-39 (Qiagen Inc.), which was spiked in as a control during RNA isolation.

#### Statistical Analysis

The study used Simon minimax two-stage design with the null hypothesis that the ORR with single-agent pembrolizumab of 24% from historical control would be tested against the combination of pembrolizumab and low-dose paclitaxel. The study was designed with a one-sided alpha of 0.09 and 90% power to detect a 50% improvement in ORR relative to historical control. The historical ORR of 24% is from a clinical trial of single-agent pembrolizumab in platinum-refractory patients with nearly identical eligibility (1). The target sample size was 27. Participant characteristics are described using descriptive statistics: medians and ranges for continuous data and frequencies for categorical data. AEs and response are summarized with frequencies. The Kaplan–Meier method was used to estimate survival and PFS. PD-L1 was categorized and the proportion of responders was calculated for each PD-L1 category. Flow cytometry and miRNA data were summarized using means and SD by best response category and time. *t* tests were used to compare these biomarkers by best response category for each timepoint. All statistical analyses were performed in SAS (v 9.4) at a two-sided alpha level of 0.05, with the exception of the exploratory biomarkers that used an alpha level of 0.15 to indicate potential differences. Analyses of biomarkers were exploratory.

Data reported here are available at clinialtrials.gov (NCT02581982) or upon request.

## Results

Twenty-seven patients were treated between April, 2016 and June 2020, with a median follow-up of 12.4 months. As shown in Table 1, the median age was 68 years (range: 49–80), with 81% men and 78% non-Hispanic White. Most patients (63%) were ECOG 1. Twenty-one of 27 (78%) had received prior definitive therapy for localized disease: chemoradiation in 24% and surgery in 76%. The majority (78%) of patients had received prior cisplatin at some point during their disease. Within 12 months of study entry, 70% of patients progressed on a cisplatin-based regimen while 30% progressed on carboplatin-based regimen.

**TABLE 1:** Patient demographics and characteristics

Baseline characteristics	All patients (*n* = 27)
Age, median (range)	68 years (49–80)
Gender, *n* (%)	
Male	22 (81%)
Female	5 (19%)
Race/ethnicity, *n* (%)	
Non-Hispanic White	21 (78%)
Black	3 (11%)
Hispanic	1 (4%)
Other/unknown	2 (7%)
ECOG performance status, *n* (%)	
0	10 (37%)
1	17 (63%)
Prior definitive therapy for localized disease, *n* (%)	21 (78%)
Chemoradiation, *n*	5
Surgery, *n*	16
Metastatic at diagnosis, *n* (%)	6 (22%)
Platinum within 12 months of study entry, *n* (%)	
Cisplatin	21 (78%)
Carboplatin	6 (22%)
Time from last chemotherapy, median (range)	124 days (14–434)
Site of metastases, *n* (%)	
Lymph node only	7 (26%)
Soft tissue, peritoneal, GU/adrenal	10 (37%)
Any visceral disease (lung and/or liver)	10 (37%)

Table 2 shows the grade 2 and higher possible, probable, or definite treatment-related AEs. Common ≥ grade 2 AEs experienced by participants were anemia (67% of participants), lymphopenia (41%), hyperglycemia (37%), and fatigue (33%). Possible treatment-related at least grade 3 or 4 AEs occurred in 56% of participants, including two immune-mediated AEs (pneumonitis and nephritis) resulting in therapy cessation but a durable PR in one case and CR in the other. There were four grade 5 events that occurred within 90 days of last treatment, all unrelated to study treatment. Two patients had documented disease progression, one was hospitalized with a gastrointestinal bleed, and one was hospitalized with a stroke. All were removed from the study and were receiving hospice care at the time of death.

**TABLE 2:** All grade 3 or higher treatment-related AEs for subjects receiving at least one dose of combination therapy. Grade 2 treatment-related AES are included if >10% frequency

		Events (*N*)			Participants (*n* = 27)
		Grade 2	Grade 3	Grade 4	*N* (%)
Immune-mediated	Pneumonitis	0	1	0	1 (3.7%)
	Increased creatinine	0	1	0	1 (3.7%)
Hematologic	Anemia	41	7	2	18 (66.7%)
	Low lymphocyte count	15	10	2	11 (40.7%)
	Neutrophil count decreased	5	2	0	3 (11.1%)
	Low white blood cell count	6	4	0	2 (7.4%)
Gastrointestinal	Diarrhea	1	2	0	3 (11.1%)
	Nausea	3	0	0	3 (11.1%)
	Vomiting	1	2	0	3 (11.1%)
	Anorexia	2	1	0	3 (11.1%)
	Alanine aminotransferase increased	1	1	0	2 (7.4%)
	Aspartate aminotransferase increased	1	1	0	2 (7.4%)
	Gastrointestinal—other	0	1	0	1 (3.7%)
Endocrine	Hyperglycemia	18	10	0	10 (37.0%)
	Hyperthyroid	0	1	0	1 (3.7%)
	Hypothyroid	4	0	0	3 (11.1%)
Neurologic	Headache	0	1	0	1 (3.7%)
	Weakness	3	1	0	3 (11.1%)
	Paresthesia	0	1	0	1 (3.7%)
	Fatigue	9	2	0	9 (33.3%)
Musculoskeletal	Arthralgia	1	1	0	2 (7.4%)
	Myalgia	0	1	0	1 (3.7%)
Cardiovascular	Hypotension	0	1	0	1 (3.7%)
Cutaneous	Pruritus	0	1	0	1 (3.7%)

**FIGURE 1:**
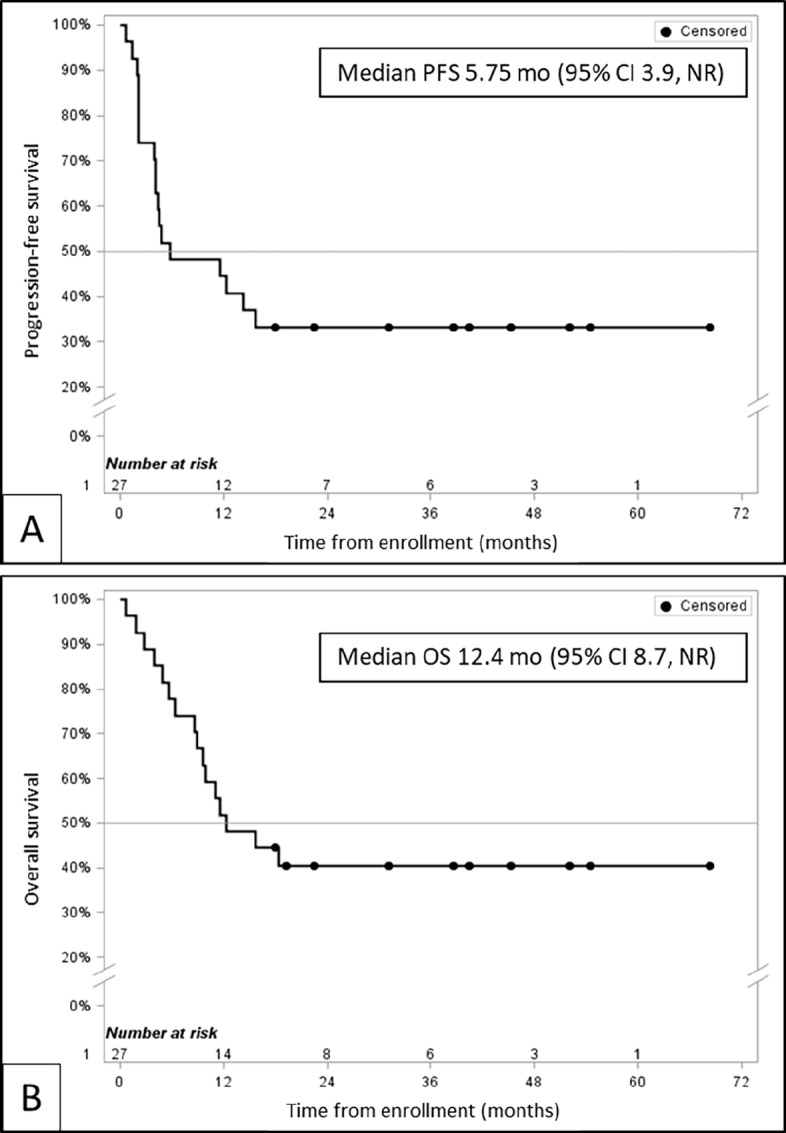
Kaplan–Meier estimates of PFS and OS. **A,** Median PFS per RECIST v1.1 is 5.75 months with 95% CI 3.9 to not reached. **B,** Median OS is 12.4 months with 95% CI 8.7 to not reached.

As shown in Table 3, the primary study endpoint of ORR by intention to treat was 9 of 27 patients (33%). Two patients had early symptomatic disease progression and moved to hospice care before subsequent imaging; therefore, ORR in patients evaluable for response by imaging was 9 of 25 (36%), including 3 with CR. Disease control rate in evaluable patients was 72%. Six-month PFS rate was 48.2% [95% confidence interval (CI): 28.7–65.2] and median OS was 12.4 months (95% CI: 8.7 months to not reached). Kaplan–Meier curves for PFS and OS are shown in Fig. 1.

**TABLE 3:** Overall response rate for all participants receiving at least one cycle of treatment and for all patients evaluable by imaging

	Best ORR: All treated *N* = 27	Best ORR: Evaluable by imaging *N* = 25	6 months response: All treated *N* = 27	6 months response: Evaluable by imaging *N* = 25
**Progressive disease (PD)**	9	7	15	13
**Stable disease (SD)**	9	9	3	3
**Partial response (PR)**	6	6	8	8
**Complete response (CR)**	3	3	1	1
**SD+PR+CR**	18; 67%	18; 72%	12; 44%	12; 48%
**PR+CR**	9; 33%	9; 36%	9; 33%	9; 36%

### Correlative Data

#### PD-L1 Expression

Evaluable tumor tissue for PD-L1 status was available for 23 of 27 patients (85%), including all 9 responding patients. Table 4 shows that there were 6 patients with no detectable PD-L1 expression, including in 3 responders. There were 5 patients with MPS > 90, including 2 responders. Evaluation of PD-L1 status in the 9 responding patients shows MPS ranging from 0 to 100, with a median of 3.

**TABLE 4:** Tissue PD-L1 status

PD-L1 status MPS	*n* = 23 *n* (%)	Best response of PR or CR (*n* = 9)	% responders
MPS 0	6 (26)	3	50%
MPS 1–9	8 (35)	3	37.5%
MPS 10–89	4 (17)	1	25%
MPS ≥90	5 (22)	2	40%

#### Immune Cell Populations

**FIGURE 2:**
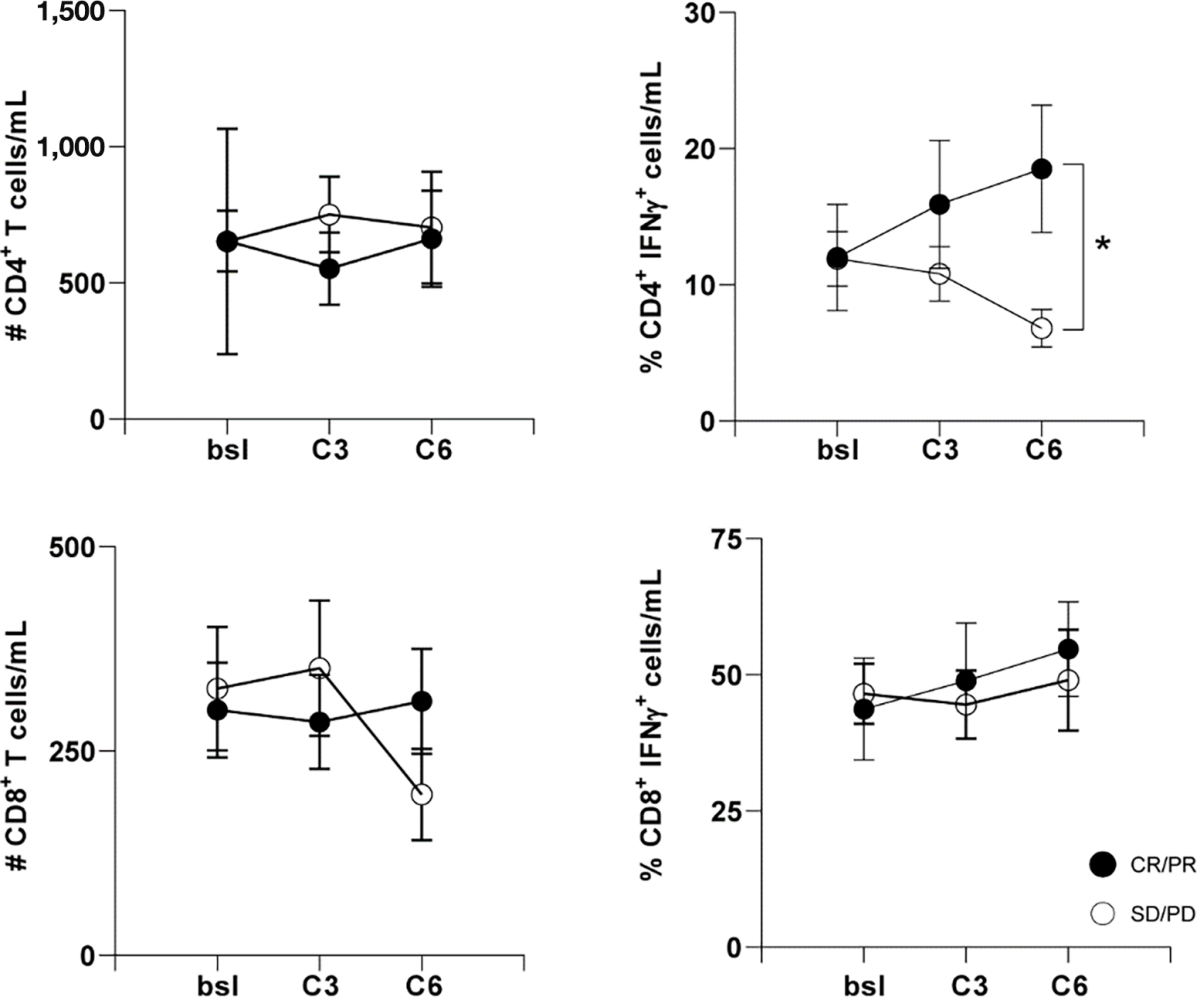
The left panels show the number of CD4^+^ T cells (top) and CD8^+^ T cells (bottom) over time, separated by responders (CR+PR, solid circles) versus nonresponders (SD+PD, open circles). The right panels show the percentage of effector T cells over time, with CD4^+^IFNγ^+^ T cells (top) and CD8^+^IFNγ^+^ T cells (bottom). Plot is mean value and error bars show ± 1 SE. The * shows the statistically significant increase in CD4^+^IFNγ^+^ T cells in responders versus nonresponders (*P* = 0.047). *X*-axis is assessment at baseline (bsl), after cycle 3 (C3), and after cycle 6 (C6).

Circulating effector CD8^+^ and CD4^+^ T cells, MDSCs, and T_REG_ cells were measured over time to determine whether there were changes in immune cell proportion or function with treatment exposure. Figure 2 shows that the number of CD8^+^ and CD4^+^ T cells did not change during treatment. Notably, however, the percentage of CD4^+^IFNγ^+^ T cells were significantly different at the end of cycle 6 in patients with CR/PR compared with patients who had SD/PD, suggesting that the percentage of circulating functional CD4^+^ T cells may correlate with response to treatment. Differences in circulating MDSC or T_REG_ populations were not observed.

#### miRNA

**FIGURE 3:**
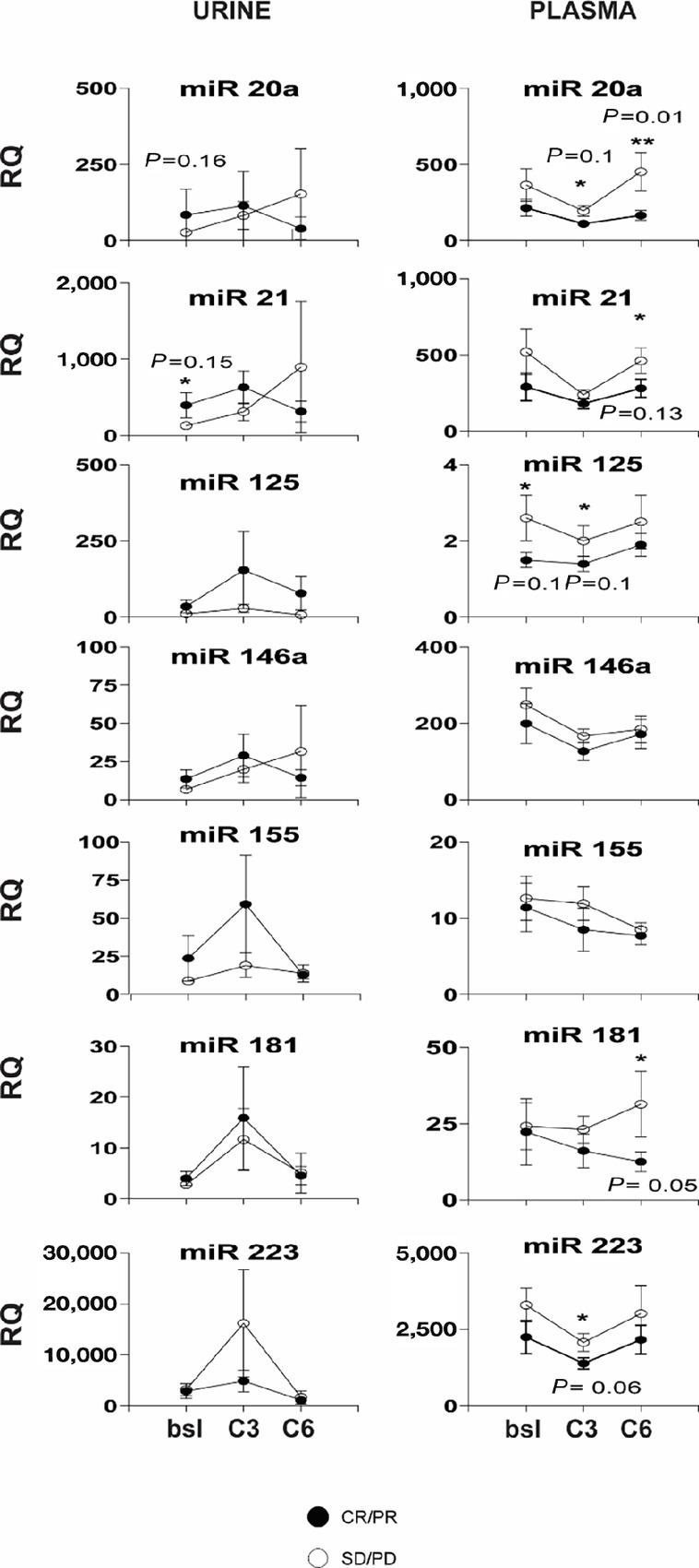
miRNAs detected at baseline, after three cycles, and after six cycles of treatment in the urine (left) and plasma (right), separated by responders (CR+PR, solid circles) versus nonresponders (SD+PD, open circles). Plot is mean value and error bars show ± 1 SE. *Y*-axis is RQ, *X*-axis is baseline (bsl), after cycle 3 (C3), and after cycle 6 (C6).

Plasma and urine levels of a panel of immunomodulatory miRNAs were compared between responders (CR/PR) and nonresponders (SD/PD) at baseline, after three cycles, and after six cycles of treatment with pembrolizumab plus paclitaxel. Figure 3 shows the differences in mean miR levels over time when graphed by best response. In general, plasma levels of immune-regulatory miRs tended to be lower in responders compared with nonresponders. Plasma miR 125 were lower in responders at baseline; miR 20a, miR 125, miR 223 were lower in responders at the end of cycle 3; and miR 20a, miR 21, miR 181 were lower in responders at the end of cycle 6, compared nonresponders. In contrast, baseline urine levels of miR 21 trended higher in responders. Although levels of urine miRs tended to be higher for responders at the ends of cycles 3 and 6, the differences were not significant. The RQ numbers for all groups are shown in Supplementary Table S1.

## Discussion

This study showed that combination therapy with low-dose paclitaxel and pembrolizumab resulted in a response rate of 33%. This response rate is higher than the 21% observed with pembrolizumab alone in the KEYNOTE-045 study in a similar platinum-refractory patient population (1). The addition of low-dose paclitaxel was well tolerated. To the best of our knowledge, this is the only study in urothelial cancer looking at the combination of low-dose chemotherapy with immunotherapy.

Despite improved outcomes with chemoimmunotherapy in other tumor types (25–28), this treatment strategy in urothelial carcinoma has yielded disappointing results. In the first-line metastatic setting, the addition of pembrolizumab to standard-dose chemotherapy did not impact OS or PFS compared with chemotherapy alone (29). Similarly, atezolizumab added to chemotherapy improved PFS but not OS (10). Recently, however, the addition of nivolumab to first-line cisplatin plus gemcitabine improves outcomes relative to chemotherapy alone (30). Furthermore, in a platinum-free first-line regimen, pembrolizumab combined with the antibody–drug conjugate EV demonstrated very good outcomes in a single-arm study (8), and outperformed chemotherapy alone in the randomized phase III EV-302 trial in terms of both PFS and OS (31). As such, the current standard-of-care for platinum-eligible patients with platinum-based chemotherapy followed by single-agent immunotherapy for maintenance (32) or at the time of disease progression (1) is likely to change moving forward. The discordant results with combination therapy across multiple studies raise many questions but further emphasize the potential benefits of chemoimmunotherapy.

Continued exploration of underlying mechanisms of response suggests that cisplatin may be immunomodulatory (33, 34). Similarly, there is both preclinical and clinical evidence that paclitaxel may activate innate immunity and therefore stimulate an inflammatory response (35). As shown here and in other studies, paclitaxel merits further study as an adjunct to checkpoint inhibition. A phase II study with pembrolizumab plus nab-paclitaxel in both platinum-ineligible and -refractory urothelial cancer showed promising results with a chemoimmunotherapy strategy (36). Another single-arm study of pembrolizumab plus nab-paclitaxel known as the PEANUT study, which was for platinum-refractory patients, demonstrated similar response rates to our study (37). Thus, three separate studies have shown a benefit of a taxane combined with pembrolizumab in refractory disease, suggesting that this strategy warrants further investigation in a randomized trial. Side effects such as alopecia and peripheral neuropathy were much less frequent in our study, suggesting a potential advantage of treating with low-dose rather than standard-dose taxane chemotherapy.

Predictive biomarkers of response to immunotherapy are urgently needed. Recent analysis of genomic data from the Cancer Genome Atlas showed that a combination of tumor mutational burden, CD8^+^ T-cell abundance, and high expression of PD-1 mRNA were the best predictors of response (38). In this study, we looked at tumor PD-L1 status, changes circulating immune cell populations, and changes in circulating and urinary miRNAs. Tumor PD-L1 status was not useful in predicting response in this study. Analysis of immune cells in the peripheral blood showed no change in MDSCs, T_REG_ cells, or CD8^+^ T cells. Interestingly, the percentage of CD4^+^IFNγ^+^ T cells increased in patients responding to treatment. While functional CD8^+^ T cells are generally correlated with antitumor responses, a recent study showed that intratumoral CD4^+^ T cells have tumor cytotoxic function in human bladder cancer (39). Human urothelial cancers upregulate expression of HLA-DR (40, 41), suggesting that cytolytic CD4^+^ T cells may be more important for antitumor responses in bladder cancer. While we did not evaluate cytolytic capacity of peripheral CD4^+^ T cells in this trial, our data provide further support for the importance of effector CD4^+^ T cells in responses to combination therapy.

Exploratory studies of blood and urine levels of a panel of immunomodulatory miRNAs as biomarkers of response were also performed. Specifically miR 155, miR 181a, and miR 20a are known to be important for T-cell differentiation and antigen response (42), miR 146a and miR 155 for natural killer (NK) cell development and function (43), miR 181a and miR 223 for NK T-cell regulation (44, 45), miR 125b, miR 146a, and miR 155 in T_REG_ cell development (46, 47), and miR 223 in MDSC regulation (48). Furthermore, miR 21 has been shown to regulate macrophage polarization and inflammatory response as well as PD-L1 expression (49–51). Although results have varied, plasma levels of several miRs have been associated with clinical outcomes in patients with melanoma, NSCLC, and renal cell carcinoma treated with immune checkpoint inhibitors (52–54). Plasma miR levels are influenced by the frequency of circulating blood cells as well as by the degree of hemolysis in the blood sample (55). How changes in circulating blood cell frequencies due to chemotherapy may influence the plasma miR results requires further study. The levels of miRs in urine, which may more directly reflect alterations in the tumor microenvironment and which is usually devoid of significant numbers of blood cells, may be a more consistent biomarker to measure. Differences in the plasma levels of miRs, including miR 181 and miR 223, between responders and nonresponders were observed in this study. Although differences in the levels of miRs, including miR 21, were also observed in urine, these were not statistically significant. Further study in larger patient populations will be necessary.

Our study was a small, single-arm, single-institution study, thus our findings can only be considered hypothesis generating. Nonetheless, the results of this study of the addition of low-dose paclitaxel to pembrolizumab in patients with platinum-refractory urothelial cancer are promising when compared with the results achieved with single-agent pembrolizumab. No unanticipated safety signals emerged. Circulating and urine biomarkers of response were also identified and warrant further study.

In conclusion, this study showed that combination pembrolizumab with low-dose paclitaxel is safe and effective in the platinum-refractory setting. Potential blood and urine biomarkers of response were also identified.

## Supplementary Material

Supplementary figure S1 The study design illustrated here shows the treatment phase with combination paclitaxel and pembrolizumab, followed by maintenance pembrolizumab if disease is stable or improved after 6 months of combination therapy.

Supplementary table S1 MicroRNA levels are shown in responders and non-responders at baseline, after 3 cycles, and after 6 cycles of treatment. P-values suggest there is a trend toward differences in plasma miR 20a, miR 21, miR 125, miR 181, and miR 223 between responders and nonresponders. In the urine, only baseline levels of miR 21 suggest a difference.
