# Supplementary material for: Low-dose Paclitaxel with Pembrolizumab Enhances Clinical and Immunologic Responses in Platinum-refractory Urothelial Carcinoma
Source: Cancer Res Commun. 2024 Feb 26;4(2):530–9. doi: 10.1158/2767-9764.CRC-23-0436 (PMC10896069; doi:10.1158/2767-9764.CRC-23-0436)
Supplement: Supplementary figure S1 — The study design illustrated here shows the treatment phase with combination paclitaxel and pembrolizumab, followed by maintenance pembrolizumab if disease is stable or improved after 6 months of combination therapy. [file crc-23-0436-s01.docx]

**Supplementary figure S1.** The study design illustrated here shows the treatment phase with combination paclitaxel and pembrolizumab, followed by maintenance pembrolizumab if disease is stable or improved after 6 months of combination therapy.

**
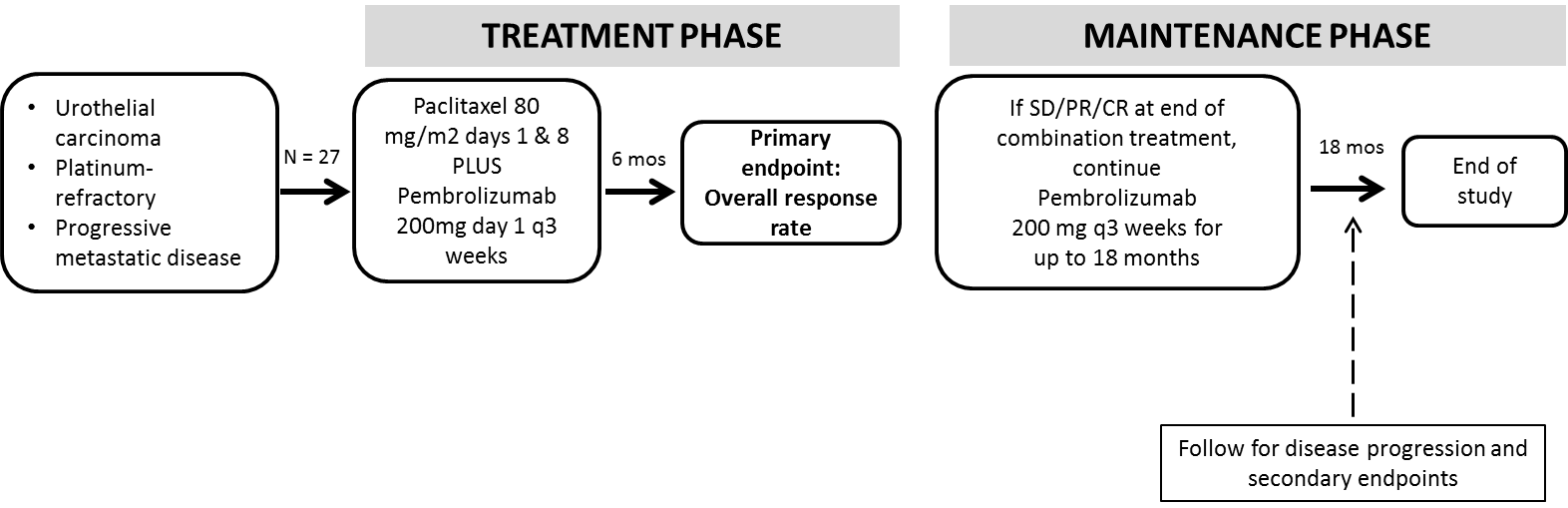
**
