# Supplementary material for: Low-dose Paclitaxel with Pembrolizumab Enhances Clinical and Immunologic Responses in Platinum-refractory Urothelial Carcinoma
Source: Cancer Res Commun. 2024 Feb 26;4(2):530–9. doi: 10.1158/2767-9764.CRC-23-0436 (PMC10896069; doi:10.1158/2767-9764.CRC-23-0436)
Supplement: Supplementary table S1 — MicroRNA levels are shown in responders and non-responders at baseline, after 3 cycles, and after 6 cycles of treatment. P-values suggest there is a trend toward differences in plasma miR 20a, miR 21, miR 125, miR 181, and miR 223 between responders and nonresponders. In the urine, only baseline levels of miR 21 suggest a difference. [file crc-23-0436-s02.docx]

**Supplementary table S1.** MicroRNA levels are shown in responders (CR/PR) and non-responders (SD/PD) at baseline, after 3 cycles (C3), and after 6 cycles (C6) of treatment. P-values suggest there is a trend toward differences in plasma miR 20a, miR 21, miR 125, miR 181, and miR 223 between responders and nonresponders. In the urine, only baseline levels of miR 21 suggest a difference.

|  | **Urine** | **CR/PR**  **Mean (SD)** | **SD/PD**  **Mean (SD)** | **p-value** | **Plasma** | **CR/PR**  **Mean (SD)** | **SD/PD**  **Mean (SD)** | **p-value** |
| --- | --- | --- | --- | --- | --- | --- | --- | --- |
| **miR 20a** | Baseline | 84.9 (108.9) | 27.2 (33.4) | 0.16 | Baseline | 214.8 (164.0) | 364.4 (413.1) | 0.23 |
|  | C3 | 114.4 (174.8) | 82.6 (168.3) | 0.67 | C3 | 110.9 (69.3) | 195.4 (137.0) | 0.10* |
|  | C6 | 39.5 (33.5) | 153.3 (209.3) | 0.58 | C6 | 166.6 (87.5) | 452.1 (217.0) | 0.01* |
| **miR 21** | Baseline | 398.1 (493.1) | 128.9 (174.3) | 0.15* | Baseline | 290.4 (273.6) | 519.3 (587.8) | 0.21 |
|  | C3 | 631.0 (656.2) | 311.3 (441.6) | 0.18 | C3 | 181 (100.8) | 239.6 (123.5) | 0.24 |
|  | C6 | 313.4 (361.3) | 896.6 (1218.7) | 0.62 | C6 | 281.3 (156.9) | 460.9 (146.9) | 0.13* |
| **miR 125** | Baseline | 35.1 (61.2) | 10.9 (12.5) | 0.27 | Baseline | 1.5 (0.7) | 2.6 (2.2) | 0.11* |
|  | C3 | 154.8 (379.8) | 29.0 (51.5) | 0.35 | C3 | 1.4 (0.5) | 2.0 (1.5) | 0.11* |
|  | C6 | 78.7 (146.3) | 7.8 (7.4) | 0.53 | C6 | 1.9 (0.9) | 2.5 (1.3) | 0.46 |
| **miR 146a** | Baseline | 13.5 (17.9) | 6.6 (6.8) | 0.30 | Baseline | 199.1 (155.9) | 248.2 (172.3) | 0.49 |
|  | C3 | 28.9 (42.0) | 19.8 (32.2) | 0.56 | C3 | 126.7 (71.1) | 166.6 (71.7) | 0.20 |
|  | C6 | 14.4 (13.9) | 31.5 (42.9) | 0.67 | C6 | 171.4 (100.3) | 184.6 (61.1) | 0.84 |
| **miR 155** | Baseline | 23.7 (44.4) | 8.9 (9.5) | 0.35 | Baseline | 11.4 (9.5) | 12.6 (11.2) | 0.80 |
|  | C3 | 59.2 (96.2) | 19.0 (29.9) | 0.25 | C3 | 8.5 (8.4) | 11.9 (8.7) | 0.36 |
|  | C6 | 12.8 (6.6) | 13.9 (8.1) | 0.84 | C6 | 7.7 (3.2) | 8.5 (1.6) | 0.72 |
| **miR 181** | Baseline | 4.0 (4.2) | 2.8 (2.7) | 0.41 | Baseline | 22.3 (32.7) | 24.2 (29.6) | 0.88 |
|  | C3 | 15.9 (30.4) | 11.7 (22.8) | 0.71 | C3 | 16.1 (16.4) | 23.1 (17.1) | 0.33 |
|  | C6 | 4.6 (4.9) | 5.1 (5.5) | 0.89 | C6 | 12.5 (8.7) | 31.4 (18.8) | 0.05* |
| **miR 223** | Baseline | 2851.5 (4485.4) | 3156.2 (4674.3) | 0.88 | Baseline | 2248.7 (1626) | 3298.5 (2136) | 0.22 |
|  | C3 | 4851.0 (6445.6) | 16223.0 (39723) | 0.31 | C3 | 1390.0 (551.9) | 2074.7 (1141) | 0.06* |
|  | C6 | 1092.0 (1325) | 1640.6 (1818.7) | 0.64 | C6 | 2165.8 (1243.8) | 3019.9 (1594.5) | 0.38 |

* p-value ≤0.15 for responders versus non-responders by t-test
